# Maternal age-related thyroid changes during pregnancy: implications for isolated hypothyroxinemia patterns

**DOI:** 10.3389/fragi.2026.1887671

**Published:** 2026-10-28

**Authors:** Saifeng Wang, Yili Zhang, Ailin Zhu, Yu Meng

**Affiliations:** 1 Department of Obstetrics and Gynecology, International Peace Maternity and Child Health Hospital, School of Medicine, Shanghai Jiao Tong University, Shanghai, China; 2 Shanghai Key Laboratory of Embryo Original Diseases, Shanghai, China

**Keywords:** adverse pregnancy outcomes, free thyroxine (FT4), isolated hypothyroxinemia, longitudinal patterns, maternal age, thyroid function

## Abstract

**Objective:**

To investigate the associations between maternal age and thyroid function in early and late pregnancy, as well as their changes, with a focus on the impact of age on the longitudinal patterns of isolated hypothyroxinemia.

**Methods:**

This retrospective cohort study included 43,545 women with singleton pregnancies. Serum free thyroxine (FT4) and thyroid-stimulating hormone (TSH) levels were measured in early and late pregnancy, along with their changes. Linear regression, restricted cubic spline (RCS), and piecewise linear regression were used to assess non-linear associations and threshold effects. Isolated hypothyroxinemia was categorized into euthyroid, transient, and persistent patterns. Ordinal logistic regression was used to examine the association between maternal age and these patterns, as well as the relationships between the patterns and adverse pregnancy outcomes, including preeclampsia, preterm birth, macrosomia, and low birth weight (LBW).

**Results:**

Maternal age showed significantly stronger negative associations with FT4 levels and their changes than with TSH. Maternal age was positively associated with the risk of isolated hypothyroxinemia in a dose–response manner. Compared with women aged 18–24 years, those aged 35–39 years (OR = 1.89) and 40–49 years (OR = 3.01) had higher risks of persistent isolated hypothyroxinemia. Risks of preeclampsia, macrosomia, and LBW increased progressively from transient to persistent patterns (all P for trend <0.01).

**Conclusion:**

Maternal age is an important factor influencing FT4 during pregnancy**.** Increasing age is associated with a higher likelihood of persistent isolated hypothyroxinemia, which is linked to a broader range of adverse pregnancy outcomes.

## Introduction

Pregnancy is accompanied by substantial changes in thyroid physiology and an increased demand for thyroid hormones (Mladenovic et al., 2025). Maternal thyroid function undergoes dynamic adaptation during pregnancy to meet the increased physiological demands of gestation and support fetal development (De Groot et al., 2012). Thyroid hormones play important roles in fetal growth and neurodevelopment (Adibi et al., 2024), while maternal thyroid dysfunction has been associated with a range of adverse pregnancy outcomes (Vamja et al., 2024). Therefore, understanding the factors that influence thyroid function during pregnancy is of considerable clinical importance. Maternal age may be an important factor influencing thyroid function during pregnancy. The prevalence of thyroid dysfunction increases with advancing age, which may reflect age-related changes in thyroid reserve and the ability to adapt to increased physiological demands (Krassas et al., 2010; Osinga et al., 2024). Meanwhile, the proportion of pregnancies at advanced maternal age has increased worldwide, partly reflecting changes in reproductive patterns and socioeconomic factors (Sparić et al., 2024). Previous studies have shown that advanced maternal age is associated with an increased risk of adverse pregnancy outcomes, including preeclampsia, preterm birth, and low birth weight (Lean et al., 2017). However, whether maternal age is associated with thyroid hormone levels during pregnancy and whether age-related differences in thyroid function are related to adverse pregnancy outcomes remain unclear. Previous studies have mainly examined thyroid dysfunction as a categorical condition or focused on thyroid function measures at a single time point during pregnancy. Evidence regarding the relationship between maternal age and thyroid function throughout pregnancy remains limited. Furthermore, it is unclear whether longitudinal patterns of thyroid dysfunction are associated with adverse pregnancy outcomes. Therefore, this study aimed to investigate the associations between maternal age and thyroid function parameters, including FT4 and TSH, during pregnancy, and to further examine the longitudinal patterns of thyroid dysfunction and their associations with adverse pregnancy outcomes.

## Methods

### Study design and population

This retrospective cohort study was conducted at a tertiary hospital in Shanghai. Medical records of women who received complete prenatal care and delivered between 2013 and 2016 were retrieved. The study protocol was approved by the ethics committee, and informed consent was waived due to the use of anonymized data from routine clinical practice. A total of 47,179 pregnant women with available early- and late-pregnancy thyroid function data were initially screened for inclusion. Multiple pregnancies (n = 950), use of thyroid-related medications before or during pregnancy (n = 1,232), stillbirths or no-live born child (n = 38), and pre-existing thyroid disease prior to pregnancy (n = 1,414) were sequentially excluded, resulting in a final study population of 43,545 women. The detailed inclusion and exclusion process is shown in Figure 1. All clinical management followed the 2012 Chinese Guidelines for the Diagnosis and Treatment of Thyroid Diseases During Pregnancy and Postpartum, which were the prevailing clinical standards during the study period.

**FIGURE 1:**
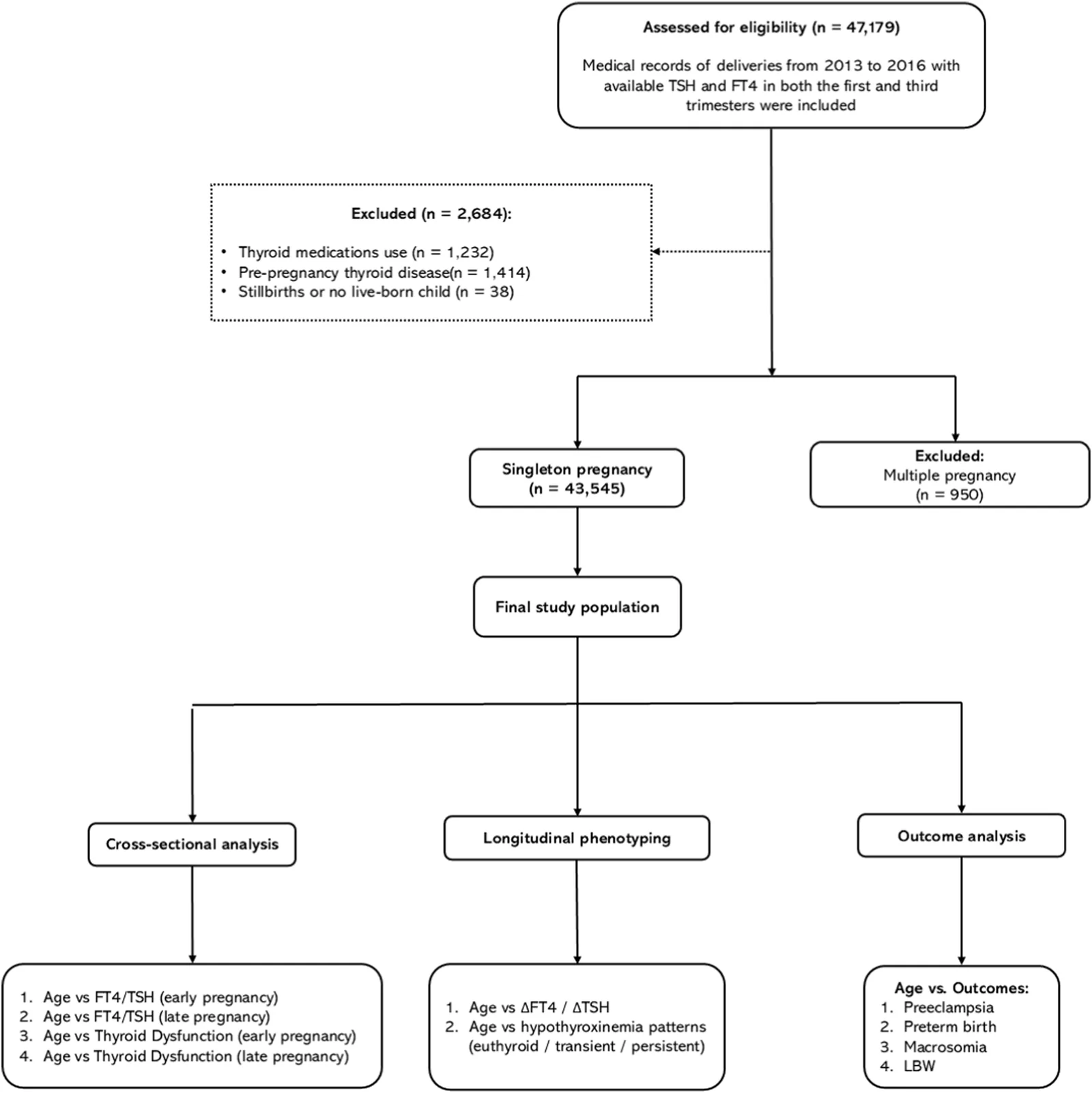
Flowchart of the study.

### Data collection

Fasting venous blood samples were collected in early pregnancy (11–13 weeks) and late pregnancy (32–34 weeks) for serum separation and measurement of thyroid-stimulating hormone (TSH), free thyroxine (FT4), and thyroid peroxidase antibodies (TPOAb) using the Architect i2000 immunoassay analyzer (Abbott, Chicago, United States) according to the manufacturer’s instructions. Blood samples were centrifuged within 6 h of collection. TPOAb ≥5.61 IU/mL was considered positive. Maternal baseline information, including age, parity, *in vitro* fertilization (IVF) conception, and education level, was collected at the first prenatal visit. Height and weight were measured to calculate pre-pregnancy body mass index (BMI). Pregnancy outcomes, such as gestational age at delivery, birth weight and other pregnancy outcomes were obtained from delivery records.

### Definition and classification criteria

The following definitions and classification criteria were used in this study based on medical guidelines and the reported literature: (a) Normal Reference Ranges of TSH and FT4: Following guidelines from the Endocrine Society and the American Thyroid Association (De Groot et al., 2012), pregnancy-specific reference ranges for early pregnancy (11–13 weeks) and late pregnancy (32–34 weeks) were established based on a local population. The 2.5th (P_2.5_) and 97.5th (P_97.5_) percentiles of TSH and FT4 were calculated to define regional reference intervals. Based on the calculated percentiles, the pregnancy-specific reference intervals were as follows: early-pregnancy FT4 11.7–19.6 pmol/L and TSH 0.03–3.64 mIU/mL; late-pregnancy FT4 9.1–14.4 pmol/L and TSH 0.39–3.67 mIU/mL. (b) Thyroid Function Categories: Thyroid dysfunction subtypes were defined as follows: overt hyperthyroidism, TSH < P_2.5_ and FT4 > P_97.5_; subclinical hyperthyroidism, TSH < P_2.5_ with FT4 within the reference range; overt hypothyroidism, TSH > P_97.5_ and FT4 < P_2.5_; subclinical hypothyroidism, TSH > P_97.5_ with FT4 within the reference range; and isolated hypothyroxinemia (IMH), FT4 < P_2.5_ with TSH within the reference range. (c) Preeclampsia: According to ACOG guidelines (Author Anonymous, 2019), preeclampsia is defined as systolic blood pressure ≥140 mmHg or diastolic blood pressure ≥90 mmHg on two occasions at least 4 h apart after 20 weeks of gestation in a previously normotensive woman, along with either 24-h urinary protein excretion ≥300 mg or a protein/creatinine ratio ≥0.3. (d) BMI categories were: Underweight (BMI <18.5 kg/m^2^), Normal weight (18.5 ≤ BMI <25 kg/m^2^), Overweight (25 ≤ BMI <30 kg/m^2^), and Obesity (BMI ≥30 kg/m^2^) (Haam et al., 2023; Jensen et al., 2014; Garvey et al., 2016). (e) Preterm Birth was defined as delivery before 37 completed weeks of gestation (Author Anonymous, 2021a). (f) Macrosomia was defined as birth weight greater than 4000 g (Author Anonymous, 2020). (g) Low birth weight (LBW) was defined as birth weight less than 2500 g (Author Anonymous, 2021b).

### Statistical analysis

Statistical analyses were performed using R software (version 4.4.1; R Core Team, 2024). Continuous variables were expressed as mean ± standard deviation (SD), and categorical variables as numbers and percentages. All statistical tests were two-sided, and P < 0.05 was considered statistically significant. Univariable and multivariable linear regression models were used to evaluate the associations between maternal age and thyroid function parameters, including FT4, TSH, ΔFT4, and ΔTSH. For late-pregnancy thyroid function and changes in thyroid function, the corresponding early-pregnancy measure was additionally included as a covariate. Restricted cubic spline (RCS) models were constructed using the “rms” package to assess potential non-linear associations, with the number and locations of knots selected based on the Akaike information criterion (AIC). When significant non-linearity was identified, two-piecewise linear regression models were used to evaluate potential threshold effects, and model fit was compared using a likelihood ratio test. Multivariable binary logistic regression models were used to examine the associations between maternal age and thyroid dysfunction categories, including overt hyperthyroidism, subclinical hyperthyroidism, overt hypothyroidism, subclinical hypothyroidism, and isolated hypothyroxinemia, with normal thyroid function as the reference group. Maternal age was analyzed as a continuous variable and categorized into five groups based on physiological characteristics, reproductive health, and common age classifications in epidemiological studies (Sheen et al., 2018; Author Anonymous, 2022; Liu et al., 2025): Young (18–24 years), Optimal (25–29 years), Borderline (30–34 years), Advanced (35–39 years), and Very Advanced (40–49 years), with the Young group as the reference. For longitudinal patterns of isolated hypothyroxinemia, multivariable binary logistic regression models were used to examine transient and persistent isolated hypothyroxinemia, with normal thyroid function throughout pregnancy as the reference group, and a P for trend was calculated. Multivariable ordinal logistic regression models were used to evaluate the associations between maternal age categories and the ordered longitudinal patterns of thyroid function (normal, transient, and persistent). Multivariable binary logistic regression models were used to evaluate the associations between longitudinal patterns of isolated hypothyroxinemia and adverse pregnancy outcomes, including preeclampsia, preterm birth, macrosomia, and low birth weight (LBW). The longitudinal pattern was additionally modeled as an ordinal variable (0 = normal, 1 = transient, and 2 = persistent) to assess potential dose-response trends. Multivariable models were adjusted for maternal age, pre-pregnancy BMI, parity, IVF conception, education level, and TPOAb status, as appropriate for each analysis, with covariates entered simultaneously. Sensitivity analyses were performed by sequentially excluding prespecified subgroups, including multiparous women, women with IVF pregnancies, women with pre-pregnancy overweight or obesity (BMI≥25 kg/m^2^), TPOAb-positive women, and women with an education level below a bachelor’s degree. Regression coefficients (β) or odds ratios (ORs) with 95% confidence intervals (CIs) were reported as appropriate.

## Results

### Quantitative assessment of the linear association between maternal age and thyroid hormone levels

To quantitatively assess the linear association between maternal age and thyroid hormone levels, univariate and multivariate linear regression models were employed to analyze the relationships between maternal age and FT4 and TSH levels in early and late pregnancy, as well as the changes in these levels during pregnancy (ΔFT4 and ΔTSH). The results are presented in Table 1. The multivariate models adjusted for several covariates, including parity, pre-pregnancy BMI, educational level, IVF and TPOAb status. To account for baseline differences in early-pregnancy FT4 and TSH, these values were included as covariates when analyzing late-pregnancy FT4 and TSH levels and changes in FT4 and TSH during pregnancy. In univariate analyses, maternal age was significantly negatively associated with both FT4 and TSH levels in early pregnancy. After adjusting for confounders, the negative association between age and early-pregnancy FT4 remained significant (β = −0.05, 95% CI: -0.06 to −0.04, P < 0.001), whereas the association with TSH was no longer significant (P = 0.970). Similarly, in univariate analyses, maternal age was negatively associated with FT4 and TSH in late pregnancy. After adjustment for confounders and early-pregnancy baseline FT4, the negative association with late-pregnancy FT4 persisted (β = −0.02, 95% CI: -0.03 to −0.02, P < 0.001), while the association with TSH was no longer significant (P = 0.993). In multivariate analyses, maternal age remained significantly negatively associated with ΔFT4 (β = −0.02, 95% CI: -0.03 to −0.02, P < 0.001), whereas no significant association was observed with ΔTSH (P = 0.981), indicating that higher maternal age is associated with a greater decline in FT4 from early to late pregnancy.

**TABLE 1:** Univariate and multivariable linear associations between maternal age and thyroid hormone levels.

Gestational period	Variables	Univariate analysis		Multivariate analysis	
		β (95% CI)	P	β (95% CI)	P
Early-pregnancy	**FT4**	−0.08 (−0.09 ∼ −0.07)	**<0.001**	−0.05 (−0.06, −0.04)	**<0.001**
	**TSH**	−0.01 (−0.99 ∼ −0.01)	**0.009**	0.00 (−0.00, 0.00)	0.970
Late-pregnancy	**FT4**	−0.04 (−0.05 ∼ −0.04)	**<0.001**	−0.02 (−0.03 ∼ −0.02)	**<0.001**
	**TSH**	−0.01 (−0.99 ∼ −0.01)	**<0.001**	0.00 (−0.00 ∼ 0.00)	0.993
From early to late pregnancy	**ΔFT4**	0.04 (0.03 ∼ 0.04)	**<0.001**	−0.02 (−0.03 ∼ −0.02)	**<0.001**
	**ΔTSH**	−0.00 (−0.00, −0.00)	0.172	−0.00 (−0.00, −0.00)	0.981

β = regression coefficient; S.E, standard error; t = t statistic; P = P value; 95% CI, 95% confidence interval. FT4 = free thyroxine; TSH, thyroid-stimulating hormone. Δ indicates the difference between late-pregnancy and early-pregnancy values. Multivariable linear regression was adjusted for education level, IVF, parity, pre-pregnancy BMI, and TPOAb. For late-pregnancy thyroid function and ΔFT4/ΔTSH, analyses, baseline FT4 and TSH, values were additionally adjusted. P values <0.05 are shown in bold type.

### Nonlinear associations between maternal age and thyroid hormone levels

The results of the multivariate linear regression analyses showed that maternal age was significantly negatively associated with FT4 levels in early and late pregnancy, while no independent linear association was observed with TSH levels. Considering that linear models may not fully capture the true shape of these relationships, we further applied RCS regression models to explore the dose–response relationships between maternal age and thyroid hormone levels, as well as potential non-linear trends. The results of the RCS analyses are presented in Figure 2. The multivariate RCS models adjusted for the same covariates as in the linear regression analyses. The number and placement of spline knots were determined based on the Akaike information criterion (AIC) to achieve an optimal balance between model fit and complexity. RCS analyses demonstrated that maternal age was significantly negatively associated with FT4 levels overall (overall effect P < 0.001). A significant non-linear negative association was observed for early-pregnancy FT4 (non-linearity P = 0.018), whereas no significant non-linear trend was found for late-pregnancy FT4 or the change in FT4 during pregnancy, with a non-linearity P value of 0.085 for both, indicating that the relationship was primarily linear. In contrast, TSH levels showed significant non-linear associations with maternal age (non-linearity P < 0.05 for all comparisons). Specifically, the overall association between age and early-pregnancy TSH did not reach statistical significance (overall effect P = 0.058). However, for late-pregnancy TSH and changes in TSH during pregnancy, age exhibited a significant U-shaped relationship (overall effect P = 0.003), with TSH levels higher at both ends of the age distribution and lowest in the mid-age range.

**FIGURE 2:**
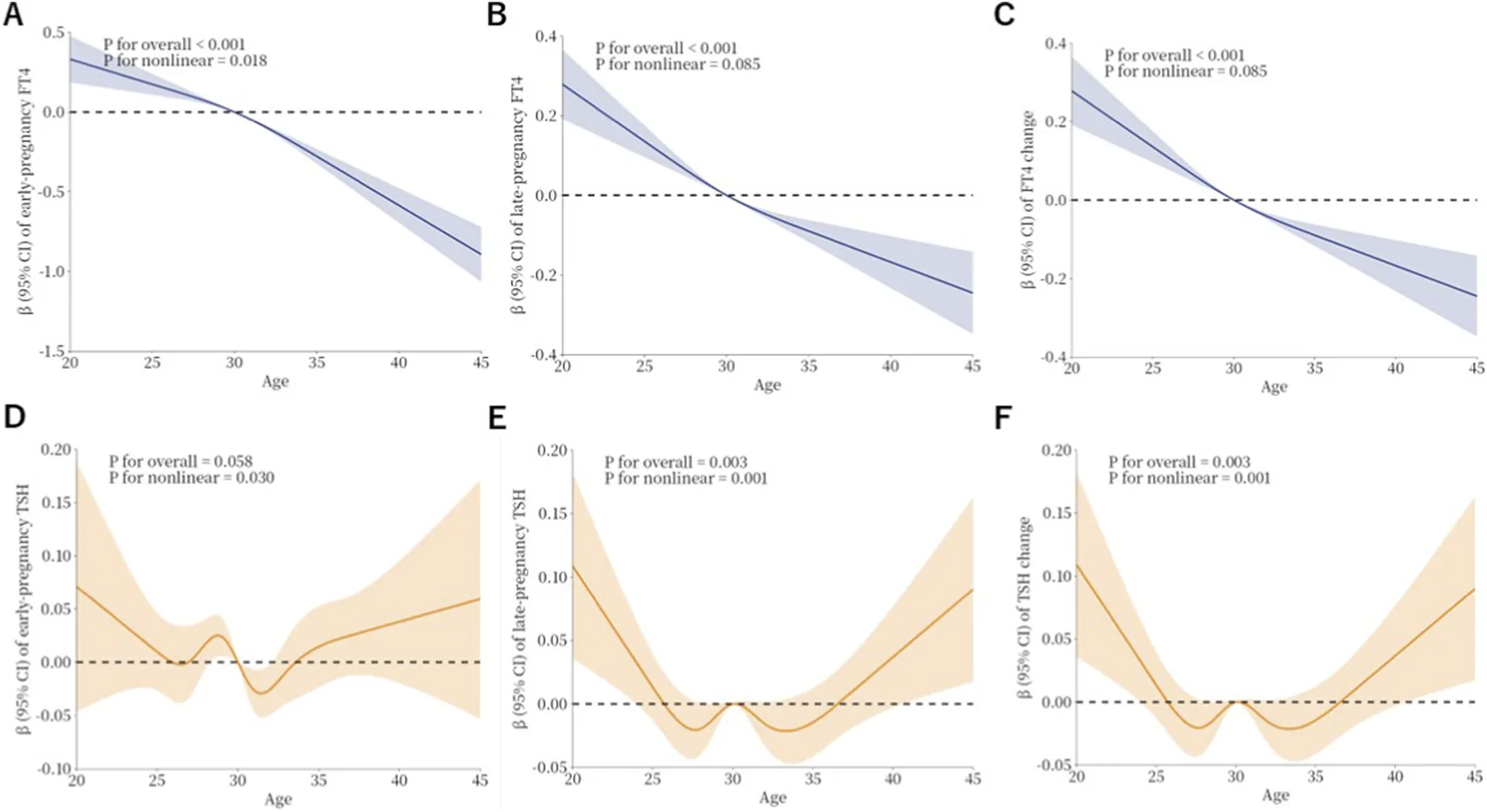
Restricted cubic spline (RCS) analyses showing the nonlinear associations between maternal age and thyroid function parameters during pregnancy. **(A–C)** show β coefficients (95% confidence intervals) for early- and late-pregnancy FT4 levels and FT4 changes. **(D–F)** display β coefficients (95% confidence intervals) for early- and late-pregnancy TSH levels and TSH changes. The solid lines represent estimated effects, with shaded areas indicating 95% confidence intervals. Dashed horizontal lines correspond to β = 0 (no effect). P-values for overall associations and tests for nonlinearity are shown in each panel. Multivariable linear regression was adjusted for education level, IVF, parity, pre-pregnancy BMI, and TPOAb.

### Threshold effects of maternal age on thyroid function during pregnancy

Based on the above RCS analyses, significant non-linear associations were observed between maternal age and early-pregnancy FT4, late-pregnancy TSH, and ΔTSH, with both overall and non-linearity P values <0.05. To further quantify potential threshold effects, segmented linear regression models were applied. The results (Table 2) identified significant age inflection points for early-pregnancy FT4, late-pregnancy TSH, and ΔTSH. Specifically, the age inflection point for early-pregnancy FT4 was 32 years. Maternal age <32 years was significantly negatively associated with early-pregnancy FT4 (β = −0.04, 95% CI: -0.05 to −0.02, P < 0.001), while the negative association was more pronounced for age ≥32 years (β = −0.07, 95% CI: -0.09 to −0.05, P < 0.001), indicating an accelerated decline in FT4 after this age. The age inflection point for late-pregnancy TSH was 25 years: for age ≤25 years, maternal age was significantly negatively associated with late-pregnancy TSH (β = −0.04, 95% CI: -0.07 to −0.01, P = 0.030), whereas no significant association was observed for age >25 years (P = 0.394). The age inflection point for ΔTSH was 27 years: maternal age ≤27 years was significantly negatively associated with ΔTSH (β = −0.02, 95% CI: -0.03 to −0.00, P = 0.008), but no significant association was found for age >27 years (P = 0.249). These findings indicate that the associations between maternal age and thyroid function indicators are both marker-specific and age-dependent. Early-pregnancy FT4 declines more rapidly after 32 years, suggesting a potential reduction in thyroid reserve capacity at this age. In contrast, the association between maternal age and late-pregnancy TSH was observed only in younger women aged 25 years or below, whereas the association with ΔTSH was limited to those aged 27 years or below. No significant associations were found in women of advanced maternal age (≥35 years). Based on these results, TSH did not demonstrate a relationship with age in the target population, which led us to focus subsequent analyses on early- and late-pregnancy FT4 levels, changes in FT4 during pregnancy, and the risk of hypothyroxinemia.

**TABLE 2:** Age threshold effects on thyroid function: Two-piecewise linear regression results.

Variable	Inflection points of age (y)	Segment	Effect (95% CI)	P-value	P for likelihood test
Early-pregnancy FT4	32	<32	−0.04 (−0.05, −0.02)	**<0.001**	**0.021**
		≥32	−0.07 (−0.09, −0.05)	**<0.001**	
Late-pregnancy TSH	25	≤25	−0.04 (−0.07, −0.00)	**0.030**	**0.008**
		>25	0.00 (−0.00, 0.00)	0.394	
TSH change	27	≤27	−0.02 (−0.03, −0.00)	**0.008**	**0.006**
		>27	0.00 (−0.00, 0.00)	0.249	

FT4, free thyroxine; TSH, thyroid-stimulating hormone; TSH, change, change in TSH, from early to late pregnancy; CI, confidence interval. Threshold effects were analyzed using piecewise linear regression models. Inflection points were identified using a data-driven recursive algorithm and rounded to the nearest integer. Effect estimates represent the regression coefficient (β) with 95% CI, for each segment. The P for likelihood test compares the goodness-of-fit between the single-line linear model (Model 1) and the two-piecewise linear model (Model 2). A significant P value (<0.05) indicates that the piecewise model provides a significantly better fit, supporting the presence of a threshold effect. P values <0.05 are shown in bold type.

### Maternal age and the risk of thyroid dysfunction during pregnancy

In the previous analyses, maternal age was significantly negatively associated with FT4 levels and FT4 changes during pregnancy. It remained unclear whether these hormone changes corresponded to clinically defined thyroid dysfunctions, including overt hyperthyroidism, overt hypothyroidism, subclinical thyroid disorders, and hypothyroxinemia. To address this, multivariate logistic regression models were applied, treating each type of thyroid dysfunction as a binary outcome to further evaluate the association between maternal age and the risk of thyroid dysfunction during pregnancy. The models adjusted for the same covariates as in the linear regression analyses to ensure consistency. In early pregnancy, the cohort included 39,885 women with normal thyroid function, 520 with overt hyperthyroidism, 60 with overt hypothyroidism, 565 with subclinical hyperthyroidism, 1,025 with subclinical hypothyroidism, and 944 with hypothyroxinemia. An additional 546 women did not meet criteria for any of these categories. In late pregnancy, 39,615 women had normal thyroid function, 113 had overt hyperthyroidism, 29 had overt hypothyroidism, 938 had subclinical hyperthyroidism, 1,026 had subclinical hypothyroidism, and 853 had hypothyroxinemia; 971 women were not classified into any of these categories. Detailed results are presented in Table 3. The results showed that, in both early and late pregnancy, the risk of isolated hypothyroxinemia increased significantly with maternal age (early pregnancy OR = 1.63, 95% CI: 1.36–1.94; late pregnancy OR = 1.63, 95% CI: 1.33–2.00). In contrast, no significant associations were observed between maternal age and other types of thyroid dysfunction, including both overt and subclinical forms of hyperthyroidism and hypothyroidism.

**TABLE 3:** Multivariate logistic regression analysis of maternal age and risk of thyroid dysfunction during pregnancy.

Gestational period	Thyroid dysfunction	n (%)	β	S.E	Z	OR (95% CI)	P
Early-pregnancy	Overt hyperthyroidism	520 (1.2%)	−0.14	0.22	−0.62	0.87 (0.57–1.34)	0.533
	Overt hypothyroidism	60 (0.1%)	0.24	0.40	0.59	1.27 (0.58–2.77)	0.555
	Subclinical hyperthyroidism	565 (1.3%)	0.01	0.02	0.50	1.01 (0.98 ∼ 1.04)	0.619
	Subclinical hypothyroidism	1,025 (2.4%)	−0.01	0.01	−0.71	0.99 (0.97 ∼ 1.02)	0.475
	Isolated hypothyroxinemia	944 (2.2%)	0.49	0.09	5.38	1.63 (1.36–1.94)	**<0.001**
Late-pregnancy	Overt hyperthyroidism	113 (0.3)	0.14	0.39	0.35	1.15 (0.53–2.48)	0.727
	Overt hypothyroidism	29 (0.1)	−0.36	0.77	−0.47	0.70 (0.15–3.12)	0.636
	Subclinical hyperthyroidism	938 (2.2)	0.03	0.13	0.27	1.03 (0.81–1.33)	0.786
	Subclinical hypothyroidism	1,026 (2.4)	−0.24	0.14	−1.76	0.78 (0.60–1.03)	0.078
	Isolated hypothyroxinemia	853 (2.0)	0.49	0.10	4.68	1.63 (1.33–2.00)	**<0.001**

β, regression coefficient; S.E, standard error; Z, Z statistic; P, P value; OR, odds ratio; CI, confidence interval. The reference group comprised women with normal thyroid function (euthyroid in early pregnancy, n = 39,885; euthyroid in late pregnancy, n = 39,615). Multivariable logistic regression models were adjusted for maternal education, IVF, status, parity, pre-pregnancy BMI, and TPOAb. P values <0.05 are shown in bold type.

### Maternal age and longitudinal patterns of isolated hypothyroxinemia

Multivariate logistic regression analyses indicated that maternal age was an independent risk factor for isolated hypothyroxinemia during pregnancy, with older women showing a significantly higher risk. Clinically, the evolution of hypothyroxinemia during pregnancy was heterogeneous: some women exhibited isolated hypothyroxinemia only in early pregnancy and returned to normal in late pregnancy (transient isolated hypothyroxinemia), whereas others had isolated hypothyroxinemia in both early and late pregnancy (persistent isolated hypothyroxinemia). These different longitudinal patterns may have distinct clinical implications and determinants. To investigate whether maternal age was associated with the longitudinal patterns of hypothyroxinemia, multivariate logistic regression models were constructed using women with normal thyroid function throughout pregnancy as the reference group. The models examined the associations between age and the risks of transient and persistent isolated hypothyroxinemia, and assessed trends in risk with increasing age (p for trend). Covariates adjusted in these models were the same as in the previous analyses. The results are presented in Table 4. Compared with women with normal thyroid function, maternal age was independently and positively associated with the risks of both transient and persistent isolated hypothyroxinemia. Specifically, the risk of transient isolated hypothyroxinemia increased with age (OR = 1.52, 95% CI: 1.24–1.86, p < 0.001), and the risk of persistent isolated hypothyroxinemia increased even more markedly (OR = 2.13, 95% CI: 1.43–3.17, p < 0.001). Trend analyses demonstrated that, with increasing maternal age, isolated hypothyroxinemia was increasingly likely to manifest as the persistent rather than the transient type (p for trend <0.001).

**TABLE 4:** Univariate and multivariate logistic regression analysis of maternal age and risk of isolated hypothyroxinemia patterns during pregnancy.

Variables	Univariate analysis					Multivariate analysis					
	β	S.E	Z	P	OR (95%CI)	β	S.E	Z	P	OR (95%CI)	p for trend*
Euthyroid	1.00 (Reference)					1.00 (Reference)					**<0.001**
Transient isolated Hypothyroxinemia	0.88	0.09	10.04	**<0.001**	2.40 (2.03 ∼ 2.85)	0.42	0.10	4.07	**<0.001**	1.52 (1.24 ∼ 1.86)	
Persistent isolated Hypothyroxinemia	0.92	0.18	5.13	**<0.001**	2.51 (1.77 ∼ 3.57)	0.76	0.20	3.73	**<0.001**	2.13 (1.43 ∼ 3.17)	

OR, odds ratio; CI, confidence interval; β, regression coefficient; S.E, standard error; Z, Z statistic.

* P for trend, test for trend across age. Euthyroid group was used as the reference category. Multivariable logistic regression models were adjusted for maternal education, IVF, status, parity, pre-pregnancy BMI, and TPOAb. P values <0.05 are shown in bold type.

### Maternal age categories and risk of isolated hypothyroxinemia

To evaluate the impact of maternal age on isolated hypothyroxinemia, participants were categorized into five age groups: Young (18–24 years, n = 1,494), Optimal (25–29 years, n = 19,359), Borderline (30–34 years, n = 17,446), Advanced (35–39 years, n = 4,682), and Very Advanced (40–49 years, n = 564). Age group was treated as an independent variable, and the longitudinal patterns of isolated hypothyroxinemia (Euthyroid, Transient isolated hypothyroxinemia, and Persistent isolated hypothyroxinemia) were treated as the ordered outcome variable in subsequent ordinal logistic regression analyses (Table 5). Baseline characteristics of the five age groups are shown in Supplementary Table S1. Multivariate models adjusted for potential confounders to estimate the independent effects of maternal age on the longitudinal patterns of isolated hypothyroxinemia. In univariate analyses, compared with the Young group, women in the Borderline, Advanced, and Very Advanced groups were more likely to show a greater tendency for the longitudinal pattern of isolated hypothyroxinemia to shift from normal to transient or persistent states, whereas the Optimal group did not differ significantly from the Young group. In multivariate analyses, the Advanced (OR = 1.89, 95% CI: 1.10–3.25, P = 0.022) and Very Advanced (OR = 3.01, 95% CI: 1.59–5.67, P < 0.001) groups remained significantly more likely to progress toward more persistent longitudinal patterns, whereas the Optimal and Borderline groups showed no statistically significant differences compared with the Young group. Overall, maternal age was associated with a dose-dependent risk of progressing to persistent isolated hypothyroxinemia, particularly among women over 35 years.

**TABLE 5:** Association between maternal age categories and risk of isolated hypothyroxinemia by ordered logistic regression.

Age category	Age Range (y)	n (%)	Univariable analysis					Multivariable analysis				
			β	S.E	t	P	OR (95% CI)	β	S.E	t	P	OR (95% CI)
Young	18–24	1,494 (3.4%)	1.00 (Reference)					1.00 (Reference)				
Optimal	25–29	19,359 (44.5%)	0.09	0.25	0.35	0.727	1.09 (0.67 ∼ 1.76)	−0.05	0.27	−0.20	0.843	0.95 (0.56 ∼ 1.61)
Borderline	30–34	17,446 (40.1%)	0.61	0.24	2.51	**0.012**	1.84 (1.14 ∼ 2.96)	0.34	0.27	1.27	0.203	1.41 (0.83 ∼ 2.37)
Advanced	35–39	4,682 (10.8%)	1.17	0.25	4.70	**<0.001**	3.22 (1.98 ∼ 5.25)	0.63	0.28	2.29	**0.022**	1.89 (1.10 ∼ 3.25)
Very advanced	40–49	564 (1.3%)	1.72	0.29	5.87	**<0.001**	5.57 (3.14 ∼ 9.88)	1.10	0.32	3.40	**<0.001**	3.01 (1.59 ∼ 5.67)

The longitudinal patterns of T4 status (Euthyroid, Transient Isolated Hypothyroxinemia, and Persistent Isolated Hypothyroxinemia) were used as an ordered dependent variable in the ordered logistic regression. Multivariable Analysis was adjusted for parity, IVF, TPOAb, maternal education level, and pre-pregnancy BMI. n (%) indicates the number of participants and the corresponding percentage in each category. P values <0.05 are shown in bold type.

### Sensitivity analyses

To assess the robustness of the association between maternal age and isolated hypothyroxinemia during pregnancy, a series of prespecified sensitivity analyses were performed. Multivariate logistic regression models were repeated after sequentially excluding multiparous women, IVF pregnancies, women with pre-pregnancy overweight or obesity (BMI ≥25 kg/m^2^), TPOAb positive individuals, and those with an education level below a bachelor’s degree, with all models adjusting for the same covariates as in the main analyses. The results are presented in Supplementary Table S2. In all sensitivity analyses, the positive association between maternal age and early- and late-pregnancy isolated hypothyroxinemia remained statistically significant (all P < 0.001). These findings indicate that the observed association between maternal age and isolated hypothyroxinemia during pregnancy is robust and not substantially influenced by the above maternal characteristics, demographic factors, or lifestyle behaviors.

### Association between different patterns of isolated hypothyroxinemia and adverse pregnancy outcomes

To evaluate the associations between longitudinal patterns of isolated hypothyroxinemia during pregnancy and adverse pregnancy outcomes, women with normal thyroid function throughout pregnancy were used as the reference group. Binary logistic regression models were applied to examine the associations between longitudinal patterns of isolated hypothyroxinemia and preeclampsia, preterm birth, macrosomia, and LBW, with normal thyroid function as the reference group (Table 6). All models adjusted for the same covariates as in the main analyses. To evaluate dose-dependent associations with adverse outcomes, isolated hypothyroxinemia status was modeled as an ordinal variable (euthyroid, transient, persistent), and P for trend was calculated. Compared with women with normal thyroid function, the risk of preeclampsia was significantly higher in the transient isolated hypothyroxinemia (OR = 1.87, 95% CI: 1.25–2.81, P = 0.002) and the persistent low T4 group (OR = 2.56, 95% CI: 1.29–5.11, P = 0.007), with a significant increasing trend as the isolated hypothyroxinemia pattern progressed toward persistence (P for trend = 0.008). The risk of macrosomia was also elevated in both the transient (OR = 1.76, 95% CI: 1.38–2.26, P < 0.001) and persistent low T4 groups (OR = 2.26, 95% CI: 1.41–3.63, P < 0.001), with a significant dose–response trend (P for trend = 0.002). For preterm birth, no significant association was observed in the transient isolated hypothyroxinemia (OR = 0.98, P = 0.901), whereas the persistent low T4 group had a significantly increased risk (OR = 1.99, 95% CI: 1.12–3.53, P = 0.019); however, the overall trend test did not reach statistical significance (P for trend = 0.062). Regarding LBW, the transient isolated hypothyroxinemia showed no significant association (OR = 0.77, P = 0.441), while the persistent isolated hypothyroxinemia had a significantly increased risk (OR = 2.60, 95% CI: 1.20–5.62, P = 0.015), with a significant overall trend (P for trend = 0.038).

**TABLE 6:** Association between different patterns of isolated hypothyroxinemia and adverse pregnancy outcomes.

Outcome	Group	Incidence (%)	Univariate analysis		Multivariate analysis		
			P	OR (95%CI)	P	OR (95%CI)	*P for trend
Premature	Euthyroid	4.2	1.00 (Reference)		1.00 (Reference)		0.062
	Transient isolated hypothyroxinemia	4.3	0.328	1.19 (0.84 ∼ 1.69)	0.901	0.98 (0.66 ∼ 1.44)	
	Persistent isolated hypothyroxinemia	8.6	**0.004**	2.26 (1.30 ∼ 3.92)	**0.019**	1.99 (1.12 ∼ 3.53)	
Preeclampsia	Euthyroid	2.0	1.00 (Reference)		1.00 (Reference)		**0.008**
	Transient isolated hypothyroxinemia	4.2	**<0.001**	2.34 (1.62 ∼ 3.38)	**0.002**	1.87 (1.25 ∼ 2.81)	
	Persistent isolated hypothyroxinemia	5.9	**0.001**	3.05 (1.55 ∼ 5.99)	**0.007**	2.56 (1.29 ∼ 5.11)	
Macrosomia	Euthyroid	6.0	1.00 (Reference)		1.00 (Reference)		**0.002**
	Transient isolated hypothyroxinemia	12.4	**<0.001**	2.05 (1.62 ∼ 2.58)	**<0.001**	1.76 (1.38 ∼ 2.26)	
	Persistent isolated hypothyroxinemia	14.5	**<0.001**	2.50 (1.59 ∼ 3.94)	**<0.001**	2.26 (1.41 ∼ 3.63)	
LBW	Euthyroid	2.2	1.00 (Reference)		1.00 (Reference)		**0.038**
	Transient isolated hypothyroxinemia	1.6	0.831	0.94 (0.53 ∼ 1.67)	0.441	0.77 (0.39 ∼ 1.50)	
	Persistent isolated hypothyroxinemia	5.3	**0.016**	2.56 (1.19 ∼ 5.49)	**0.015**	2.60 (1.20 ∼ 5.62)	

LBW, low birth weight. Euthyroid pregnant women were used as the reference group. The multivariable model was adjusted for maternal age, pre-pregnancy BMI, parity, IVF, conception, TPOAb, status, and education level.

* P for trend was calculated by entering low T4 status as an ordinal variable (0 = euthyroid, 1 = transient isolated hypothyroxinemia, 2 = persistent isolated hypothyroxinemia) in the model. P values <0.05 are shown in bold type.

In summary, persistent isolated hypothyroxinemia was significantly associated with preeclampsia, preterm birth, macrosomia, and LBW. Transient isolated hypothyroxinemia was associated only with preeclampsia and macrosomia. Overall, the risks of preeclampsia, macrosomia, and LBW showed a significant increasing trend with progression from normal thyroid function to transient and persistent isolated hypothyroxinemia, indicating a dose–response relationship.

## Discussion

This study systematically evaluated the association between maternal age and thyroid function during pregnancy and identified three main findings. First, in the cross-sectional analyses, maternal age was significantly and inversely associated with FT4 levels in both early and late pregnancy, independent of maternal parity, pre-pregnancy BMI, IVF conception, education level, and TPOAb status. Second, the longitudinal analyses demonstrated that maternal age was associated not only with FT4 levels but also with changes in FT4 during pregnancy and the persistence of isolated hypothyroxinemia. Advanced maternal age was associated with a greater decline in FT4 from early to late pregnancy and a higher likelihood of persistent rather than transient isolated hypothyroxinemia. Third, distinct longitudinal patterns of isolated hypothyroxinemia were significantly associated with adverse pregnancy outcomes, with persistent isolated hypothyroxinemia showing greater clinical impact. Collectively, these findings suggest that maternal age is associated not only with absolute FT4 levels during pregnancy but also with longitudinal change in FT4 and the persistence of thyroid dysfunction.

In the cross-sectional analyses, maternal age was significantly and inversely associated with FT4 levels in both early and late pregnancy, and this association remained significant after adjustment for relevant maternal characteristics. These findings are consistent with those of most previous studies and support the concept that thyroid functional reserve gradually declines with advancing age (Jansen et al., 2024). Among women of advanced maternal age, FT4 may be a more sensitive marker than TSH for detecting age-related changes in thyroid function. From a physiological perspective, aging may affect both the hypothalamic–pituitary–thyroid (HPT) axis and the thyroid gland itself. Age-related structural and functional changes in the thyroid, including smaller follicular epithelial cells, reduced colloid content, and decreased gland volume with increased density, may impair thyroid hormone synthesis and secretion (Puzianowska-Kuźnicka, 2013). In addition, Carlé et al. reported an attenuated hypothalamic–pituitary response to low thyroid hormone levels in older individuals, which may reduce the sensitivity of TSH to age-related declines in peripheral thyroid function (Carlé et al., 2007). Together, these mechanisms may explain why FT4, rather than TSH, better reflect age-related changes in thyroid function in our study.

More importantly, our longitudinal analyses further demonstrated an independent association between maternal age and changes in FT4 during pregnancy. We found that maternal age was significantly and inversely associated with ΔFT4, indicating that women of advanced maternal age experienced a greater decline in FT4 from early to late pregnancy. This finding extends the evidence from previous cross-sectional studies. It suggests that maternal age may influence the ability of the thyroid system to adapt to the increased physiological demands of pregnancy. Thyroid aging is characterized by reduced functional reserve, impaired T4 synthesis, and diminished TSH responsiveness (Franceschi et al., 2019), while previous epidemiological and physiological studies have demonstrated age-related alterations in thyroid hormone metabolism and functional reserve (Wu et al., 2018). Experimental and human studies have further supported a progressive decline in thyroid function with advancing age (Visser et al., 2016), and the responsiveness of the HPT axis to thyroid hormone deficiency may also become attenuated with age (Chaker et al., 2018; Hadlow et al., 2013). During early pregnancy, hCG stimulates the TSH receptor, thereby promoting thyroid hormone secretion and contributing to a transient increase in FT4 (Moleti et al., 2014). This adaptive response may be limited by reduced thyroid functional reserve. Thus, women of advanced maternal age may have a diminished capacity to compensate for the increased thyroid hormone requirements of pregnancy, potentially resulting in a greater decline in FT4. Age-related chronic low-grade inflammation, mitochondrial dysfunction, cellular senescence, and epigenetic alterations may further contribute to impaired thyroid hormone homeostasis (Hong et al., 2023).

Our study further demonstrated that maternal age was closely associated with the longitudinal patterns and persistence of isolated hypothyroxinemia. With increasing maternal age, women were not only more likely to develop isolated hypothyroxinemia but also more likely to exhibit a persistent rather than transient pattern. This finding may be explained by an age-related decline in the capacity for endocrine adaptation during pregnancy. Pregnancy imposes sustained physiological demands on the maternal endocrine system, requiring increased thyroid hormone production to meet maternal and fetal requirements. Younger women may have greater thyroid functional reserve and therefore be better able to restore FT4 levels later in pregnancy, whereas women of advanced maternal age may have limited compensatory capacity because of reduced thyroid reserve. Experimental evidence also suggests that aging is associated with alterations in peripheral thyroid hormone metabolism. Barnhoorn et al. reported a hypothyroid state in the liver of aging mouse models, accompanied by reduced DIO1 activity and increased DIO3 activity (Barnhoorn et al., 2023), findings that are consistent with those reported by Visser et al. (Visser et al., 2016). In addition, previous studies have shown that advanced maternal age is independently associated with an increased risk of endocrine morbidity (Rotem et al., 2026). The concept of pregnancy as an accelerator of aging further highlights the substantial physiological demands that pregnancy places on maternal endocrine, metabolic, and organ reserves (Ryan et al., 2024; Pham et al., 2024). Consistent with these observations, our ordinal regression analysis showed that, compared with women younger than 35 years, those aged ≥35 years had a significantly higher risk of persistent isolated hypothyroxinemia, with the risk increasing further among women aged >40 years. These findings suggest a potential age-related gradient in the decline of thyroid adaptive capacity during pregnancy.

In addition, the relationship between FT4 and fetal neurodevelopment has received increasing attention. Thyroid hormones are involved in multiple critical processes of fetal brain development, including neuronal proliferation, migration, differentiation, myelination, and synaptic maturation. Because fetal thyroid function is not fully established during early pregnancy, fetal development depends substantially on the maternal supply of thyroid hormones. Maternal FT4 levels may therefore be important for fetal neurodevelopment (de Escobar et al., 2004). Previous studies have suggested that lower maternal FT4 levels, particularly isolated hypothyroxinemia, may be associated with neurodevelopmental and cognitive outcomes in offspring, although the evidence has not been entirely consistent. A systematic review and meta-analysis reported an increased risk of intellectual disability in offspring, while a large prospective cohort study found that lower FT4 levels in early pregnancy were associated with lower childhood IQ and reduced gray matter volume (Thompson et al., 2018; Korevaar et al., 2016). However, a recent adolescent follow-up study found no significant associations between maternal thyroid function and most neurodevelopmental outcomes (Keestra et al., 2026). In the context of our findings, the greater decline in FT4 and higher likelihood of persistent low FT4 among women of advanced maternal age may have potential implications for fetal neurodevelopment. However, because offspring neurodevelopment was not directly assessed in our study, a causal relationship between maternal FT4 trajectories and neurodevelopment cannot be established. Further prospective studies integrating longitudinal maternal FT4 trajectories with standardized assessments of offspring neurodevelopment are warranted to investigate this possibility.

Our study further demonstrated that different longitudinal patterns of isolated hypothyroxinemia were significantly associated with adverse pregnancy outcomes. Compared with transient isolated hypothyroxinemia, persistent isolated hypothyroxinemia was associated with greater clinical impact, suggesting that the duration of low FT4 exposure may influence pregnancy outcomes. Transient reductions in FT4 mainly occur during early pregnancy, a critical period for placental development, fetal neurodevelopment, and metabolic programming. Thyroid hormones also contribute to fetal anabolic metabolism and energy allocation (Silva de Morais and Leung, 2024), and maternal thyroid dysfunction may alter fetal metabolic programming through effects on adipose tissue development, lipid deposition, and insulin sensitivity (Zhu et al., 2019), potentially contributing to the association between low FT4 and macrosomia. In addition, thyroid hormones are involved in placental angiogenesis and vascular remodeling. Experimental studies have shown that T4 promotes the expression of placental angiogenic factors such as VEGF and PlGF, whereas hypothyroidism reduces their expression and placental vascularization (Silva et al., 2015). These findings provide a potential biological explanation for the association between transient isolated hypothyroxinemia and preeclampsia observed in our study. In contrast, persistent low FT4 exposure extends throughout pregnancy and may have broader effects on fetal growth and other physiological processes, potentially increasing the risks of preterm birth and low birth weight. Consistent with this interpretation, the risk of adverse pregnancy outcomes increased progressively from transient to persistent isolated hypothyroxinemia in our study, in agreement with the findings reported by Su et al. (Su et al., 2021).

These findings suggest that maternal age may be an important consideration in the assessment of thyroid function during pregnancy. Reliance solely on uniform trimester-specific reference ranges may not fully capture age-related differences in longitudinal FT4 trajectories. In particular, isolated hypothyroxinemia detected in early pregnancy among women of advanced maternal age may warrant closer follow-up, as these women may be more likely to develop persistent isolated hypothyroxinemia. Age-stratified longitudinal FT4 trajectories may therefore help inform more individualized strategies for monitoring thyroid function during pregnancy. However, this was an observational study, and offspring neurodevelopment was not directly assessed. Therefore, the underlying mechanisms and causal relationships proposed in this study require further evaluation in prospective studies.

## Strengths and limitations

This study has several strengths. First, it is a large-scale retrospective cohort study with a sample size of 43,545 pregnant women, providing sufficient statistical power to detect subtle associations and perform robust subgroup and sensitivity analyses. Second, the availability of both early- and late-pregnancy thyroid function measurements allowed us to characterize longitudinal patterns of hypothyroxinemia (transient vs. persistent), a novel approach that distinguishes this study from most prior research that relied solely on cross-sectional data. Third, the use of advanced statistical methods (including RCS analysis to detect non-linear relationships, piecewise linear regression to identify threshold effects, and ordered logistic regression with trend tests to evaluate dose-response relationships) strengthened the rigor of our findings. Fourth, comprehensive adjustment for potential confounders and multiple sensitivity analyses demonstrated the robustness of the results. However, several limitations should be acknowledged. First, the observational design precludes causal inference. Although we identified dose-response relationships and temporal patterns, residual confounding cannot be entirely excluded. Second, thyroid function was assessed only at two time points (early and late pregnancy), limiting our ability to capture more detailed longitudinal trajectories or to precisely determine the timing of recovery in the transient group. Third, maternal iodine status, which may influence thyroid function, was not available. In addition, exclusion of stillbirths may have introduced selection bias. Fourth, this study was conducted at a single center, although the large sample size supports generalizability, multicenter validation is warranted. Finally, Pharmacological treatment for thyroid dysfunction during pregnancy may have influenced thyroid function measurements in late pregnancy, reflecting thyroid function under real-world, guideline-directed clinical management. However, none of the women with isolated hypothyroxinemia received levothyroxine treatment, consistent with the clinical guidelines in effect during the study period, thereby reducing the potential impact of treatment on its longitudinal classification.

## Data Availability

The raw data supporting the conclusions of this article will be made available by the authors, without undue reservation.
