# Supplementary material for: Maternal age-related thyroid changes during pregnancy: implications for isolated hypothyroxinemia patterns
Source: Front Aging. 2026 Oct 28;7:1887671. doi: 10.3389/fragi.2026.1887671 (PMC13751370; doi:10.3389/fragi.2026.1887671)
Supplement: Supplementary file 1 [file Table1.docx]

**Table S1. Baseline characteristics of participants by maternal age groups**

| **Variable** | | **Age (years)** | | | | |
| --- | --- | --- | --- | --- | --- | --- |
|  |  | **<25** | **25–29** | **30–34** | **35-39** | **≥40** |
| **Parity (n,%)** | **Nulliparas** | 1,433 (95.9) | 18,162 (93.8) | 13,502 (77.4) | 2,447 (52.3) | 262 (46.4) |
|  | **Multiparas** | 61 (4.1) | 1,197 (6.2) | 3,944 (22.6) | 2,235 (47.7) | 302 (53.6) |
| **IVF (n,%)** | **Without IVF** | 1,491 (99.8) | 19,173 (99.0) | 16,796 (96.3) | 4,289 (91.6) | 505 (89.5) |
|  | **With IVF** | 3 (0.2) | 186 (1.0) | 650 (3.7) | 393 (8.4) | 59 (10.5) |
| **TPOAb (n,%)** | **Negative** | 1,370 (91.7) | 17,442 (90.1) | 15,614 (89.5) | 4,204 (89.8) | 491 (87.0) |
|  | **Positive** | 124 (8.3) | 1,917 (9.9) | 1,832 (10.5) | 478 (10.2) | 73 (13.0) |
| **Maternal Education (n,%)** | **Below bachelor’s degree** | 866 (58.0) | 4,688 (24.2) | 3,243 (18.6) | 1,078 (23.0) | 143 (25.4) |
|  | **Bachelor’s degree or higher** | 628 (42.0) | 14,671 (75.8) | 14,203 (81.4) | 3,604 (77.0) | 421 (74.6) |
| **Pre-pregnancy BMI (kg/m²)** | | 20.23 ± 3.11 | 20.73 ± 2.69 | 21.25 ± 2.77 | 21.60 ± 2.73 | 21.99 ± 3.02 |
| **Early-pregnancy FT4 (pmol/L)** | | 15.49 ± 2.07 | 15.20 ± 2.01 | 14.91 ± 2.03 | 14.47 ± 2.02 | 14.12 ± 1.85 |
| **Early-pregnancy TSH (mIU/mL)** | | 1.36 ± 1.01 | 1.35 ± 0.95 | 1.32 ± 0.93 | 1.32 ± 0.91 | 1.34 ± 0.89 |
| **Late-pregnancy FT4 (pmol/L)** | | 11.89 ± 1.36 | 11.68 ± 1.39 | 11.51 ± 1.33 | 11.29 ± 1.30 | 11.19 ± 1.25 |
| **Late-pregnancy TSH (mIU/mL)** | | 1.69 ± 0.94 | 1.63 ± 0.86 | 1.60 ± 0.83 | 1.60 ± 0.82 | 1.68 ± 0.85 |
| **ΔFT4 (pmol/L)** | | -3.60 ± 1.94 | -3.52 ± 1.84 | -3.40 ± 1.83 | -3.18 ± 1.81 | -2.94 ± 1.62 |
| **ΔTSH (mIU/mL)** | | 0.33 ± 0.91 | 0.28 ± 0.80 | 0.27 ± 0.76 | 0.28 ± 0.74 | 0.34 ± 0.71 |

*Data are presented as mean ± SD for continuous variables, and n (%) for categorical variables. BMI, body mass index; IVF, in vitro fertilization; TPOAb, thyroid peroxidase antibody; FT4, free thyroxine; TSH, thyroid-stimulating hormone; Δ indicates the difference between late-pregnancy and early-pregnancy values.; SD, standard deviation. No statistical comparisons were made, as differences were adjusted for in multivariable models.*

**Table S2. Sensitivity analyses of maternal age on isolated hypothyroxinemia**

| **Variables** | **n (%)** | **Early-pregnancy** | | | **Late-pregnancy** | | |
| --- | --- | --- | --- | --- | --- | --- | --- |
|  |  | **OR** | **95%CI** | **P** | **OR** | **95%CI** | **P** |
| **All patients** | 43,545 (100.0%) | 1.09 | 1.07 ~ 1.11 | <0.001 | 1.07 | 1.05 ~ 1.10 | <0.001 |
| **Excluding multiparous women** | 35,806 (82.2%) | 1.10 | 1.08 ~ 1.13 | <0.001 | 1.07 | 1.04 ~ 1.09 | <0.001 |
| **Excluding IVF conceptions** | 42,254 (97.0%) | 1.09 | 1.07 ~ 1.11 | <0.001 | 1.07 | 1.05 ~ 1.09 | <0.001 |
| **Excluding overweight / obese individuals (BMI ≥ 25 kg/m²)** | 40,169 (92.2%) | 1.08 | 1.06 ~ 1.11 | <0.001 | 1.07 | 1.05 ~ 1.09 | <0.001 |
| **Excluding TPOAb positive individuals** | 39,131 (89.9%) | 1.09 | 1.06 ~ 1.11 | <0.001 | 1.07 | 1.05 ~ 1.10 | <0.001 |
| **Excluding maternal education level below bachelor’s degree** | 33,527 (77.0%) | 1.09 | 1.06 ~ 1.11 | <0.001 | 1.07 | 1.04 ~ 1.09 | <0.001 |

*IVF, in vitro fertilization; TPOAb, thyroid peroxidase antibody. Multivariable logistic regression was used to estimate β coefficients and 95% confidence intervals (CI). Models were adjusted for parity, IVF, TPOAb, maternal education level, and pre-pregnancy BMI. In the analysis of late-pregnancy hypothyroxinemia, early-pregnancy FT4 was included as an additional covariate*
